# Analysis of Different Binding Modes for Tiagabine within the GAT-1 Transporter

**DOI:** 10.3390/biom12111663

**Published:** 2022-11-09

**Authors:** Kamil Łątka, Marek Bajda

**Affiliations:** Department of Physicochemical Drug Analysis, Faculty of Pharmacy, Jagiellonian University Medical College, Medyczna 9, 30-688 Cracow, Poland

**Keywords:** GABA transporters, GAT-1, tiagabine, binding mode

## Abstract

The recently obtained cryo-electron microscopy structure (PDB code: 7SK2) of the human γ-aminobutyric acid transporter type 1 (hGAT-1) in complex with the antiepileptic drug, tiagabine, revealed a rather unexpected binding mode for this inhibitor in an inward-open state of the transporter. The simultaneously released crystal structures of the modified dopamine transporter with mutations mimicking hGAT-1 indicated an alternative binding mode for the tiagabine analogues that were found to block the transporter in an outward-open state, which is more consistent with the results of previous biological and molecular modeling studies. In view of the above discrepancies, our study compares different hypothetical tiagabine binding modes using classical and accelerated molecular dynamics simulations, as well as MM-GBSA free binding energy (dG) calculations. The results indicate that the most stable and energetically favorable binding mode of tiagabine is the one where the nipecotic acid fragment is located in the main binding site (S1) and the aromatic rings are arranged within the S2 site of the hGAT-1 transporter in an outward-open state, confirming the previous molecular modelling findings. The position of tiagabine bound to hGAT-1 in an inward-open state, partially within the intracellular release pathway, was significantly less stable and the dG values calculated for this complex were higher. Furthermore, analysis of the cryo-electron map for the 7SK2 structure shows that the model does not appear to fit into the map optimally at the ligand binding site. These findings suggest that the position of tiagabine found in the 7SK2 structure is rather ambiguous and requires further experimental verification. The identification of the main, high-affinity binding site for tiagabine and its analogues is crucial for the future rational design of the GABA transporter inhibitors.

## 1. Introduction

Tiagabine is an antiepileptic drug used in the treatment of partial seizures [1]. Its pharmacological mechanism of action is based on the selective inhibition of gamma-aminobutyric acid (GABA) transporters type 1 (GAT-1), resulting in increased levels of this inhibitory neurotransmitter within the GABAergic synapses [2,3]. In addition to GAT-1, there are three other types of GABA transporter, BGT-1, GAT-2, and GAT-3, which differ in their cellular and anatomical distribution [3]. GABA transporters belong to the SLC6 protein family, which exploits the concentration gradient of sodium and chloride ions to co-transport substrates across the cell membrane [4]. The SLC6 family also includes other important therapeutic targets, such as monoamine transporters, inhibitors of which have been used for years in the therapy of depression and anxiety, as well as obsessive-compulsive or eating disorders [4]. Insights into the structure of the transporters from the SLC6 family and their mechanism of interaction with ligands were possible due to the release of the crystal structure of the bacterial transporter for leucine (aLeuT), and then successively also the structures of the dopamine transporter from *Drosophila melanogaster* (dDAT) and the human serotonin (hSERT) and glycine transporters (hGlyT-1) [5,6,7,8,9]. These proteins consist of 12 transmembrane domains with TM1-TM5 and TM6-TM10 related by a pseudo-two-fold-axis. In the half-length of the TM1 and TM6 domains, there are non-helical fragments that, together with the adjacent TM3, TM8, and TM10 domains, form the main binding site (S1) where substrates and ions bind. During the transport cycle, three main conformational states of transporters can be distinguished: an outward-open state, allowing for access to the S1 site for the substrate and ions from the extracellular side; an occluded state, where access to the S1 site is blocked from both sides of the membrane; and an inward-open state, allowing for the release of the substrate and ions into the cell [10,11]. Analysis of the available crystal structures of SLC6 proteins and the results of in silico studies revealed that competitive inhibitors bind with a high affinity to the S1 site, blocking the transporter in an outward-open state [6,7,12,13,14,15,16]. Within the vestibule of the transporter, an allosteric binding site (S2) was identified, in which, as observed for S-citalopram in SERT, additional ligand molecules can bind, thus modulating the affinity of ligands (including itself) that bind in the S1 site [8,17,18]. Inhibitors displaying fully non-competitive kinetics appeared to block the transporter in an outward-open state, as in the case of vilazodone located within the vestibule of the SERT, or in an inward-open state, as seen for ibogaine (SERT inhibitor) and the bitopertin analogue (GlyT-1 inhibitor) [9,11,19,20,21]. Molecular modelling studies based on the homology models of GAT-1 indicated that tiagabine bound partially within the S1 site (nipecotic acid fragment, mimicking the substrate) and partially in the S2 site (3-methylthiophene rings), stabilizing an outward-open state of the transporter [22,23,24,25,26]. This binding mode correlated well with the results of the cysteine scanning mutagenesis studies of GAT-1 and the competitive/mixed-type (depending on the assay conditions) inhibition displayed by tiagabine [27,28,29,30,31,32]. A breakthrough in investigating the structure of GABA transporters was the recent cryo-electron microscopy structure of the human GAT-1 transporter in a complex with tiagabine (PDB code: 7SK2) [33]. It confirmed the high structural similarity of GAT-1 with the other SLC6 transporters, but revealed a rather unexpected binding mode of tiagabine, which was found to block GAT-1 in an inward-open state. While the nipecotic acid fragment of tiagabine was located within the S1 site, the aromatic fragments of the inhibitor were arranged within the intracellular release pathway, similarly to those from the bitopertin analogue in GlyT-1. To bind in this way, tiagabine needs to diffuse across the cell membrane and reach the binding site from the intracellular side, or to bind from the extracellular side and then squeeze through the middle of the transporter, as suggested by Motiwala et al. Interestingly, in parallel with obtaining the cryo-electron microscopy structure of hGAT-1, Joseph et al. released the crystal structure of DAT with modifications at the S1 site mimicking hGAT-1 (DAT_GAT_), in complex with tiagabine analogues NO711 and SKF89976a (PDB codes: 7WGT, 7WLW) [34]. In the crystal structure of DAT_GAT_, compounds NO711 and SKF89976a are located entirely at the S1 site, blocking the transporter in an outward-open state. An additional molecule of SKF89976a was found at the S2 site, which seems to explain its mixed-type kinetics of inhibition. Although the crystal structure of DAT_GAT_ does not completely reflect the wild type of hGAT-1, it presents the possible alternative binding mode of tiagabine within hGAT-1 compared with that observed in the 7SK2 structure. In view of the discrepancies in the recent findings, in this work, we compared the tiagabine binding modes discussed above using molecular docking, molecular dynamics, and MM-GBSA binding free energy calculations. In addition, the cryo-electron microscopy structure 7SK2 was re-analyzed to verify its reliability. Our study aimed to indicate the main, high-affinity binding mode of tiagabine and its analogues, which is important for the future rational design of new inhibitors of GABA transporters.

## 2. Results

**Figure 1:**
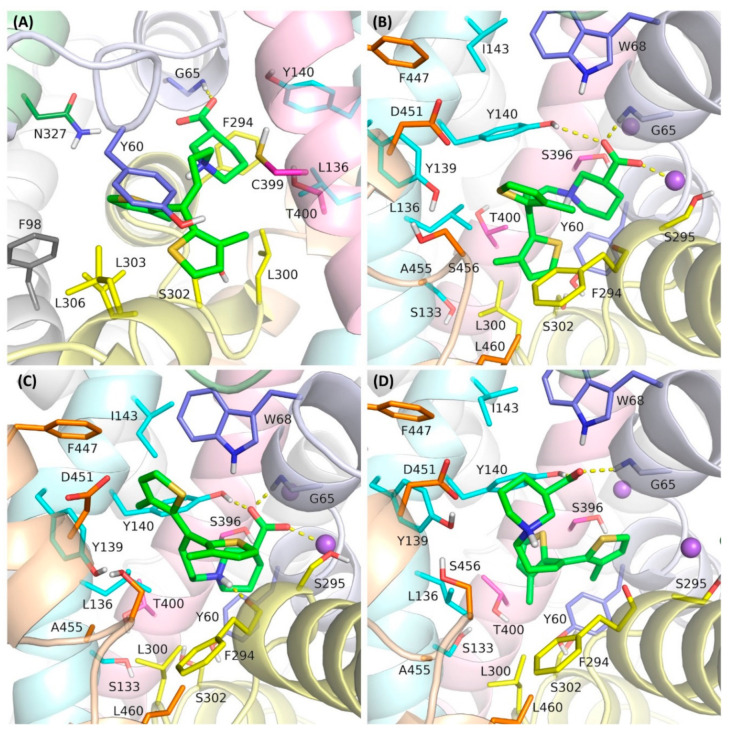
Investigated binding modes of tiagabine within hGAT-1: (**A**) mode 1, derived from 7SK2 structure; (**B**) mode 2, corresponding to the position of the tiagabine analogue in the DAT_GAT_ crystal structures; (**C**) mode 3, pose obtained in molecular modeling studies using a homology model of hGAT-1 based on the 4XP9 template; (**D**) mode 4, reflecting the first step of the hypothetical two-step induced fit mechanism of tiagabine transition through the GAT-1 transporter. The transmembrane domains and their constituent amino acids are colored as follows: TM1–blue violet; TM2–grey; TM3–cyan; TM6–yellow; TM7–green; TM8–pink; TM10–orange. Sodium ions are presented as purple spheres. Hydrogen bonds and ionic interactions are shown as yellow dashed lines.

In this study, we investigated four binding modes of tiagabine in hGAT-1. The first was that observed in the cryo-electron microscopy structure of 7SK2, in an inward-open state (designated as mode 1) (Figure 1A). The other complexes represented tiagabine approaching the transporter from the extracellular side, blocking GAT-1 in an outward-open state. In one of these modes, tiagabine is located entirely in the S1 site, corresponding to the arrangement of the NO711 and SKF89976a in the crystal structure of the DAT_GAT_ (designed as mode 2) (Figure 1B). This mode was obtained by docking tiagabine to the hGAT-1 homology model that was built using the 7WGT crystal structure as a template. In the third mode that was compared, the nipecotic acid fragment of tiagabine is located in the S1 site and the aromatic fragments are accommodated within the S2 site of GAT-1 (mode 3) (Figure 1C). This pose was presented in our previous work and was obtained by the docking of tiagabine to a hGAT-1 homology model based on the crystal structure of the dopamine transporter (PDB code: 4XP9) [22]. 

In the last of the studied binding modes, the aromatic rings of tiagabine are arranged at the S1 site, while the nipecotic acid fragment is located above the extracellular gate (Tyr140–Phe294 line) (mode 4) (Figure 1D). This mode reflects the first step in tiagabine binding according to the hypothesis proposed by Motiwala et al. [33]. This hypothesis suggests that tiagabine initially binds to the transporter in an outward-open state, inducing subsequent conformational changes leading to an inward-open state with the final arrangement of tiagabine, as observed in the 7SK2 structure. This complex was obtained by the docking of tiagabine to the hGAT-1 homology model built on the 4XP9 template, followed by further optimization. 

For each of the investigated binding modes of tiagabine, we performed two 100 ns classical molecular dynamics simulations (MD 1 and MD 2) in DESMOND and two 100 ns Gaussian accelerated MD (GaMD 1 and GaMD 2) in NAMD. GaMD reduces the energy barriers by smoothing the potential energy surface and thus provides a more than 30 times speedup of the occurring conformational changes relative to the classical dynamics. It enables observing possible changes in the ligand arrangement, which require considerable time [35,36].

**Figure 2:**
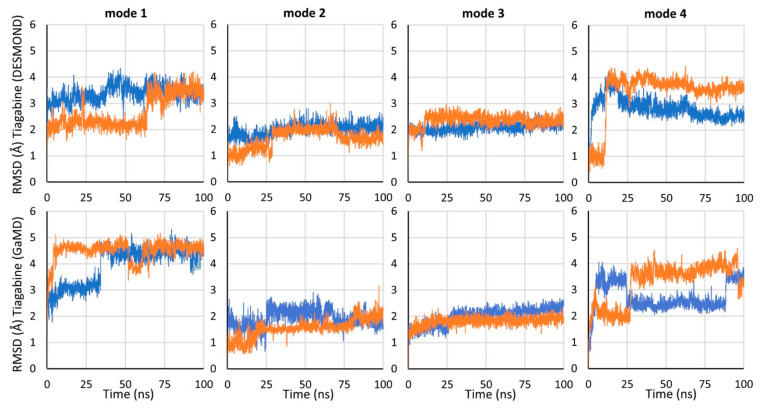
RMSD changes for tiagabine in different starting binding modes in the course of the molecular dynamics simulations carried out with DESMOND (MD) and NAMD (GaMD) program. In each graph, the RMSD for MD 1 is shown in dark blue and for MD 2 in orange.

In the simulations carried out, mode 2 and mode 3 were found to be the most stable (Figure 2, Figure S1). In all of the simulations for these modes, the carboxyl group of tiagabine formed robust hydrogen bonds with the main chain of Gly65 and the hydroxyl group of Tyr140, and coordinated the sodium ion. For mode 3, a very stable hydrogen bond was also observed between the protonated nitrogen atom of tiagabine and the main chain of Phe294. Notably, in the case of mode 2, this nitrogen atom did not appear to form any significant interactions within the S1 site of hGAT-1. Regarding the arrangement of the aromatic rings of tiagabine in mode 2, in the simulations performed with DESMOND, they shifted towards the S2 site, which was particularly emphasized in MD 1. This weakened the hydrophobic interactions with Leu300 and Tyr60, lining the S1 site while enhancing those with Tyr140 from the extracellular gate, and Ile143 and Trp68 located in the S2 site. In the case of GaMD simulations, the aromatic fragments retained their position (GaMD 1) or moved slightly towards Leu300 from the non-helical fragment of TM6 (GaMD 2); however, they did not reach as deep into the S1 site as observed for tiagabine analogues in the 7WGT and 7WLW crystal structures. It should be noted here that the modifications in DAT_GAT_ resembling hGAT-1 did not involve TM10. In hGAT-1, there is a non-helical fragment in half of the length of TM10 caused by the presence of one additional amino acid in the hGAT-1 sequence. This non-helical fragment is close to the site where the aromatic rings of tiagabine and its analogues bind in mode 2, limiting the space available for their accommodation. Moreover, this site is further reduced in hGAT-1 by the presence of Leu460 (TM10) in place of Ile483 in DAT_GAT_. This may explain why the aromatic rings of tiagabine were not located as deep in the main binding site of hGAT-1 as in DAT_GAT_, and why in some simulations, they moved towards the vestibule of the transporter. For mode 3, the final arrangement of 3-methylthiophene rings was very similar in all four simulations (Table 1). One of the rings rotated from its starting position and the whole lipophilic fragment of tiagabine moved slightly down towards the S1 site, but still occupying the allosteric site and preserving most of the initially formed hydrophobic interactions, mainly with the side chains of Trp68, Tyr140, Tyr139, Phe294, and Ile143. Additionally, a beneficial CH–π stacking with Tyr140 was observed. 

In mode 4, the tiagabine behaved significantly less stably and there were greater differences in its final arrangement when comparing the programs used and MD repetitions (Figure 1, Table 1). During simulations performed with DESMOND, the aromatic rings remained entirely in the main binding site, forming hydrophobic interactions mainly with Tyr60, Thr400, Leu300, and Tyr140. The hydrogen bond between the carboxyl group of tiagabine and the side chain of Tyr140 was retained in only one simulation (MD 1). In this case, the possibility of a hydrogen bond with Gly65 and the coordination of the sodium ion was also observed. In MD 2, the carboxyl moiety of tiagabine shifted towards the side chain of Arg69, forming a salt bridge with this residue. An analogous interaction with Arg69 could be observed in accelerated simulations performed in NAMD. In only one of the GaMD simulations for mode 4, a significant interaction involving the protonated nitrogen atom of the nipecotic acid fragment was identified. This was a hydrogen bond with the main chain of Ala455 from the non-helical fragment of TM10. In GaMD simulations for mode 4, only one of the 3-methylthiophene rings remained in the S1 site. The other was located at the level of the extracellular gate (Tyr140–Phe294 line), forming hydrophobic and π–π interactions with Tyr140. The arrangement of the tiagabine found in the cryo-electron microscopy structure of 7SK2 (mode1) was also unstable in the molecular dynamics simulations (Figure 1). Although the final poses of tiagabine did not differ drastically between GaMD 1 and GaMD 2, and were very consistent for repetitions of DESMOND simulations (Table 1), there was a large discrepancy when comparing the results from the two programs and the relatively high RMSD values calculated versus the initial pose. The largest deviations concerned the aromatic fragment of tiagabine. In the MD simulations performed with DESMOND, it shifted towards the TM8 and TM3 domains, forming hydrophobic interactions with the side chains of Thr400, Leu300, and Leu137 within the S1 site. Meanwhile, the hydrophobic and aromatic interactions with the Tyr60 were significantly impaired. In the GaMD simulations, the aromatic fragment of tiagabine shifted towards the non-helical fragment of TM1, thus enhancing the hydrophobic interactions with the side chain of Phe98 from TM2, while remaining unaffected (GaMD 1) or weakened (GaMD 2) interactions with Tyr60. The carboxyl group of tiagabine in the 7SK2 complex generally retained its position and interactions, of which the hydrogen bond with Gly65 was the most robust. However, no significant stable interactions created with the participation of the protonated amine moiety of the nipecotic acid fragment were found. It is noteworthy that in the 7SK2 complex, we could observe the largest changes in the position of the amino acids around the ligand. This is particularly pronounced for the amino acids forming TM1a and TM6b, which interacted with the aromatic fragment of tiagabine (Supplementary Figure S2). It should be mentioned here that Motiwala et al. performed a series of 1 µs classical molecular dynamics simulations for the 7SK2 complex, using the Gromacs software. The authors indicated that most of the MD simulations confirmed the stability of the tiagabine binding mode found in the 7SK2 structure, which appeared to differ from the results obtained in our study, particularly with regard to the stability of the aromatic fragments of tiagabine. These discrepancies may result from the application of different approaches or the analysis of results. Although the shorter dynamics simulations described herein might be perceived as a limitation, the 100 ns accelerated GaMD simulations enable, as mentioned above, tracking conformational transitions requiring even more than the 3 µs of the classical MD simulations. It should also be noted that the 7SK2 complex was not compared with other hypothetical tiagabine binding modes in terms of the stability and binding energy. The poses of tiagabine after MD simulations were also not described precisely, and only the distances between the nearest heavy atoms of the individual amino acids (no distinction between side chains and backbone) and tiagabine in a particular MD frames were presented. This does not fully illustrate the spatial arrangement of the particular tiagabine fragments and GAT-1 residues, which is important for hydrogen bonds and aromatic interactions. Therefore, it is difficult to compare in detail the results obtained in our study with those obtained by Motiwala et al.

**Figure 3:**
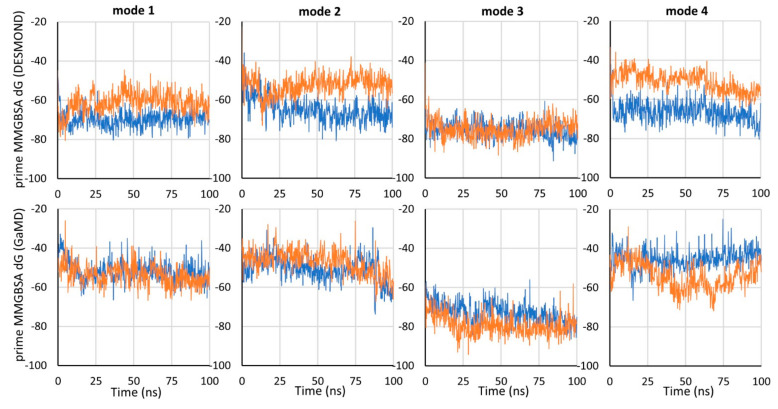
MM-GBSA binding free energy values (kcal/mol) for tiagabine in different binding modes in the course of the molecular dynamics simulations carried out with the DESMOND (MD) and NAMD (GaMD) programs. In each graph, the RMSD for MD 1 is shown in dark blue and for MD 2 it is in orange.

To compare the binding energy of tiagabine in the investigated complexes with hGAT-1, the MM-GBSA binding free energy (dG) was calculated using the Prime program for the frames of each simulation. Mode 3 was proven to be the most energetically beneficial, which is particularly evident when comparing the GaMD simulations (Figure 3). For mode 3, dG values were also the most consistent between simulations performed with NAMD and DESMOND. For the 7SK2 complex (mode 1), and modes 2 and 4, lower dG values were obtained for simulations performed with DESMOND (in both or one of the simulations). 

Taking into account the obtained RMSD and free binding energy values, mode 3 of tiagabine binding within hGAT-1 appears to be the most reliable and displays the highest affinity. Significantly, only in mode 3 did we observe a stable, reproducible interaction formed with the participation of the protonated nitrogen atom of tiagabine (hydrogen bond with the main chain of Phe294). The lack of specific interactions in the other modes might be alarming because such a group is significant for an activity on the transporter, and it seems that it should be involved in the binding. Although in our study we only applied in silico methods, some data from previous biological experiments seem to support our results.

Firstly, as mentioned in the introduction, kinetics studies clearly indicate that tiagabine as well as its analogues exhibit a competitive/mixed type of inhibition, and its binding is dependent on sodium ions [32,33,34,37,38]. Ibogaine and bitopertin analogue, which were reported previously to bind in an inward-open state of SERT and GlyT-1, respectively, are fully non-competitive inhibitors [9,11,20,39]. In addition, bitopertin as well as its analogues have a more lipophilic structure enriched with fluorine atoms, which enables them to diffuse through the cell membrane much more easily than the relatively more hydrophilic tiagabine molecule [40,41]. It seems even less likely that tiagabine reaches the binding site observed in the 7SK2 structure by passing through the main binding site of hGAT-1. Whereas nipecotic acid is a substrate that competes with GABA, tiagabine is not transported by GAT-1 [32,42]. It is also quite unlikely that the aromatic rings of tiagabine enter the S1 site of the transporter before the nipecotic acid fragment, as suggested by Motiwala et al. The nipecotic acid fragment that mimics the substrate is expected to reach the S1 site first, attracting the rest of the ligand. This is confirmed by the significantly higher stability and more favorable energy obtained for mode 3 than mode 4. It is noteworthy that in none of the simulations for modes 2, 3, or 4, we observed TM1a bending and other conformational changes suggesting a transition of the transporter into an inward-open state. Furthermore, ibogaine, while blocking SERT in an inward-open state, remains located at the S1 site, not entering deeper into the intracellular release pathway as observed for tiagabine in the 7SK2 structure. This is interesting taking into consideration that ibogaine is a smaller molecule than tiagabine, and SERT is adapted to transport serotonin, which is significantly larger than GABA. The rationale for the binding of tiagabine in an outward-open state is provided by the results of reactivity studies of GAT-1 cysteine mutants for sulfhydryl reagents. The presence of tiagabine analogues enhances reactivity to the non-penetrating sulfhydryl reagents of cysteine residues replacing the amino acids lining the vestibule of the transporter (Lys448, Tyr452, Tyr453 from TM10; Asp287, Leu286, and Thr290 from TM6; Tyr72 from TM1), which are accessible to the extracellular environment only in an outward-open state [27,28,31]. Interestingly, there was reduced reactivity of hGAT-1 mutants in which cysteine was introduced in place of amino acids that, according to mode 3, are in close proximity to the aromatic rings of tiagabine, i.e., Ser456, Ser295, Trp68, and Leu64. Moreover, tiagabine analogues decreased reactivity to penetrating sulfhydryl reagents of cysteine residues replacing amino acids lining the intracellular release pathway, i.e., Cys399, Thr406, and Thr402, accessible to the intracellular environment in an inward-open state [29,30]. Of the listed amino acids, only Cys399 is close enough to the position of tiagabine from the 7SK2 structure, to be directly protected by this molecule. Thr406 and Thr402 are located at a substantial distance from bound tiagabine, and their reduced reactivity can only be explained by the outward-open state of the transporter. The opposite results were obtained for ibogaine. Its presence increased the reactivity of cysteine residues introduced in positions of amino acids that are more exposed to the intracellular environment in an inward-open state (Ser91, Ser277, Val281, Ser347, Ala441, and Thr448) and showed a protective effect of those replacing residues accessible to the extracellular environment in an outward-open state (Ser404 and Tyr107) [20,43].

**Figure 4:**
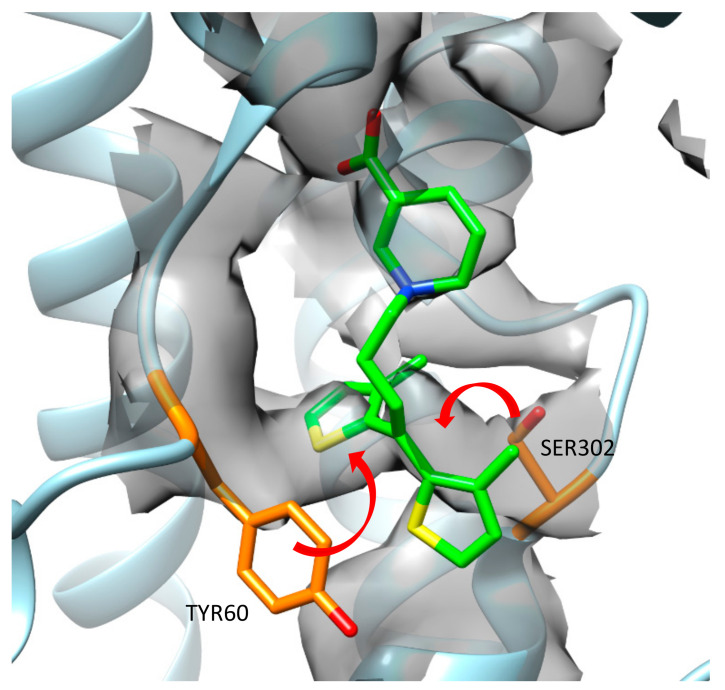
Possible arrangement of Tyr60 and Ser302 which improve the fit of them into the cryo-EM map.

During an analysis of the published cryo-EM map and 7SK2 structure, it was observed that some amino acid residues from the model did not fit into the map very well. Moreover, they could occupy the area where one of the thiophene rings from tiagabine was placed. If side chains of Tyr60 and Ser302 were moved, they would interact with each other, creating a hydrogen bond (Figure 4). In such a case, the binding mode of tiagabine should be different, which confirms our in silico hypothesis that tiagabine interacts with GAT-1 mainly in an outward-open state. 

## 3. Conclusions

In this study, we investigated four hypothetical binding modes of tiagabine in the hGAT-1 transporter, using classical molecular dynamics and Gaussian accelerated molecular dynamics (GaMD) simulations in DESMOND and NAMD, respectively. Additionally, the MM-GBSA binding free energy values were calculated for the tiagabine-hGAT-1 complexes obtained in each simulation. The most stable binding mode was found to be the one in which the nipecotic acid fragment of tiagabine was located in the main binding site (S1) and the aromatic rings were arranged within the S2 site of hGAT-1, blocking the transporter in an outward-open state. The carboxyl group of tiagabine formed substrate-mimicking interactions with the sodium ion, Gly65, and the side chain of Tyr140, while the protonated amine moiety created a hydrogen bond with the main chain of Phe294. The aromatic fragment formed hydrophobic interactions mainly with Trp68, Tyr140, Tyr139, Phe294, and Ile143, and a CH–π interaction with Tyr140. The presented binding mode also displayed the most beneficial binding free energy values and was supported by the results of the kinetics data and studies with sulfhydryl reagents. The binding mode, in which tiagabine is located entirely in the S1 site of the transporter, similar to that observed for tiagabine analogues in DAT_GAT_ crystal structures, was slightly less stable and there were greater differences in the DESMOND and NAMD results. This mode also showed higher dG values, and, in addition, the protonated amine group of tiagabine was not involved in any significant interaction. A similar lack of interactions of the tiagabine amino moiety and less beneficial free binding energy were found for the 7SK2 structure. In addition, the position of tiagabine from the 7SK2 structure was significantly less stable. Only the carboxyl group of tiagabine formed robust interactions, while the aromatic fragment shifted from its initial position within the intracellular release pathway. The binding mode, in which the aromatic rings of tiagabine were located at the S1 site of the transporter and the nipecotic acid was located above the extracellular gate, was also unstable and had higher dG values. This tiagabine arrangement in an outward-open state was supposed to reflect the first step in a hypothetical two-step transition of tiagabine to an inward-open state. However, the lack of stability of this tiagabine pose and that of the 7SK2 structure, together with the absence of conformational changes indicative of a transporter transition into an inward-open state, suggest that the binding of tiagabine in an inward-open state in the manner observed in the 7SK2 structure is rather ambiguous. Furthermore, the location of the tiagabine in the 7SK2 structure requires careful consideration due to the worse fit of the model to the cryo-electron map in this key area. Our study encourages further insight into the interactions of tiagabine and other inhibitors with GABA transporters, which may help in the rational design of new ligands with desirable properties in the future.

## 4. Methods

To analyze binding mode 1 of tiagabine with MD simulations and MM-GBSA dG calculations, we used the 7SK2 structure without modifications. Other binding modes were obtained by docking tiagabine to hGAT-1 homology models. In the case of mode 2, the hGAT-1 model on the 7WGT template was built in the SWISS-MODEL server based on automatically generated alignment [44]. Sodium and chloride ions were transferred to the model directly from the template. The model was devoid of an *N-* and *C-terminus* due to the low sequence similarity between hGAT-1 and DAT_GAT_. The non-helical fragment of the TM10 domain in a model (Tyr453–Ser459) was additionally optimized in the Modeller 10.1 program using the MyModel class [45]. Among the 200 models with an optimized TM10 fragment, 10 with the best DOPEscore values were pre-selected. They were then compared with the 7SK2 structure and the one in which the conformation of the non-helical TM10 fragment was most similar to this in the 7SK2 structure was finally selected. For tiagabine binding modes 3 and 4, we used the hGAT-1 homology model built on the 4XP9 template described in our previous paper [22]. In the case of mode 3, we used a model optimized for nipecotic acid derivatives [22]. For mode 4, we employed the hGAT-1 model before optimization. Before docking, all models were prepared with the Protein Preparation Wizard available in Schrödinger Suite, using default settings. The tiagabine molecule was optimized with LigPrep in the OPLS3e force field and the ionization state was predicted with Epik v5.3 for pH = 7.4 ± 0.5.

Docking of the tiagabine molecule to the appropriate hGAT-1 models was carried out with the Glide v8.8 program using standard precision (SP) [46]. In the hGAT-1 model based on the 7WGT template (mode 2), the grid center was defined by the position of the NO711 molecule transferred from a template. For modes 3 and 4, grid was defined by the Tyr140 and Phe294 residues. The inner box size in all cases was 15 Å × 15 Å × 15 Å. Five poses of tiagabine were written out for each docking. For mode 4, the complex obtained after docking was further optimized with Refine Protein-Ligand Complex protocol available in Schrödinger Suite (Prime v6.1). The VSGB solvation model and local optimization sampling algorithm were applied. Optimization involved ligand and residues within 7 Å of ligand, excluding the sodium and chloride ions. All of the calculations were performed using the OPLS3e force field. 

The input files for the molecular dynamics in DESMOND v6.3 [47] were prepared with the System Builder module of the Schrödinger Suite. A TIP3P water type and POPC(300K) membrane model were applied. The model structure pre-aligned with the OPM server was placed in a membrane. The size of the orthorhombic box was calculated according to the buffer method. The buffer distance between the protein model and the simulation box boundary was set as follows: a = 12 Å, b = 12 Å, c = 10 Å. The system was neutralized by adding an appropriate number of chloride ions. Then, 0.15 M NaCl was added to provide the standard physiological ionic strength. MD simulations in DESMOND v6.3 were run in an NPT ensemble at 300 K with timestep of 2 fs. The total duration of each simulation was 100 ns with a recording interval of 50 ps. The system was relaxed before the simulation according to the default six-step DESMOND protocol. A seed was set as random and the other options were default. During the system setups as well as the simulations, the OPLS3e force field was applied.

The input files for GaMD simulations in NAMD 2.13 [48] were prepared with the CHARMM-GUI server using the enhanced sampler option [49]. All hGAT-1 models were positioned in a membrane using the OPM server. For each complex, a water pore was generated. All of the complexes were embedded in a POPC membrane and solvated with TIP3P water molecules. The system size (X, Y) was set as 100 Å × 100 Å. The systems were neutralized and the standard physiological ionic strength was obtained by adding chloride ions and 0.15 M NaCl. Before the GaMD simulations, systems were equilibrated via the six-step protocol suggested for NAMD. The next two stages involved a short conventional MD and equilibration step, which aimed to collect the potential statistics needed for the actual GaMD simulations. GaMD simulations in NAMD 2.13 were run in an NPT ensemble at 303.15 K with a timestep of 2 fs and a total duration of 100 ns. The interval for both the energy and trajectory recording was 10 ps. The CHARMM36m force field was applied.

The results of the MD simulations were analyzed in the VMD 1.9.3 program. The RMSD values for heavy atoms of the tiagabine and protein were calculated after superimposing all of the frames onto the start frame according to the backbone (C, CA, and N) of the transmembrane domains surrounding the binding site, i.e., TM1, TM2, TM3, TM6, TM7, TM8, and TM10. The RMSD values for the protein were calculated taking into account both the main and side chains of the amino acids located within a distance of 7 Å from the initial tiagabine pose. RMSD values for the heavy atoms of the final tiagabine poses between the MD simulation repetitions and the programs used were calculated for 100 pairs of frames representing the last 5 ns of the compared dynamics. 

The MM-GBSA binding free energies were calculated for tiagabine-hGAT-1 complexes obtained from 500 frames of each dynamic (one frame for every 0.2 ns) using the Prime MM-GBSA v3.0 program. The VSGB solvation model and OPLS3e force field were applied for this purpose. Protein flexibility, water molecules, and additional sodium and chloride ions were ignored in the calculations. 

An analysis of the cryo-EM map and 7SK2 structure was performed with UCSF Chimera 1.16. The map was visualized using contour level 0.11 and step 1, and shown for 5 Å around the proposed tiagabine position.

## Figures and Tables

**Table 1:** RMSD values for the final poses of tiagabine from various runs of molecular dynamics simulations.

Compared MD Simulations	RMSD for Tiagabine (Last 5 ns of Simulations)			
	Mode 1	Mode 2	Mode 3	Mode 4
NAMD GaMD 1 vs. NAMD GaMD 2	2.35	1.65	1.06	2.70
DESMOND MD 1 vs. DESMOND MD 2	1.31	2.35	1.11	3.13
NAMD GaMD 1 vs. DESMOND MD 1	5.31	2.73	0.96	4.16
NAMD GaMD 1 vs. DESMOND MD 2	5.29	1.54	0.77	3.78
NAMD GaMD 2 vs. DESMOND MD 1	5.46	3.05	0.98	3.76
NAMD GaMD 2 vs. DESMOND MD 2	5.31	2.55	1.05	3.12

Table cells colored according to RMSD value: red–high RMSD values, green–low RMSD values.

## Data Availability

PDB files with the investigated complexes and movies from the molecular dynamics simulations are openly available in FigShare repository at DOI: 10.6084/m9.figshare.21463053.

## Supplementary Materials

The following are available online at https://www.mdpi.com/article/10.3390/biom12111663/s1, Figure S1: Investigated binding modes of tiagabine within hGAT-1, before and after MD simulations., Figure S2: RMSD changes for hGAT-1 residues within 7 Å of tiagabine in different binding modes during molecular dynamics simulations carried out with DESMOND (classical MD) and NAMD (GaMD) programs.
