# Supplementary material for: Analysis of Different Binding Modes for Tiagabine within the GAT-1 Transporter
Source: Biomolecules. 2022 Nov 9;12(11):1663. doi: 10.3390/biom12111663 (PMC9687605; doi:10.3390/biom12111663)
Supplement: Supplementary file 1 [file biomolecules-12-01663-s001.zip › biomolecules-1981353-supplementary.pdf]

biomolecules

MDPI

Supplementary information

Analysis of Different Binding Modes for Tiagabine within the GAT-1 Transporter

Kamil Łątka and Marek Bajda *

Department of Physicochemical Drug Analysis, Faculty of Pharmacy, Jagiellonian University Medical College, Medyczna 9, 30-688 Cracow, Poland
* Correspondence: Correspondence: marek.bajda@uj.edu.pl

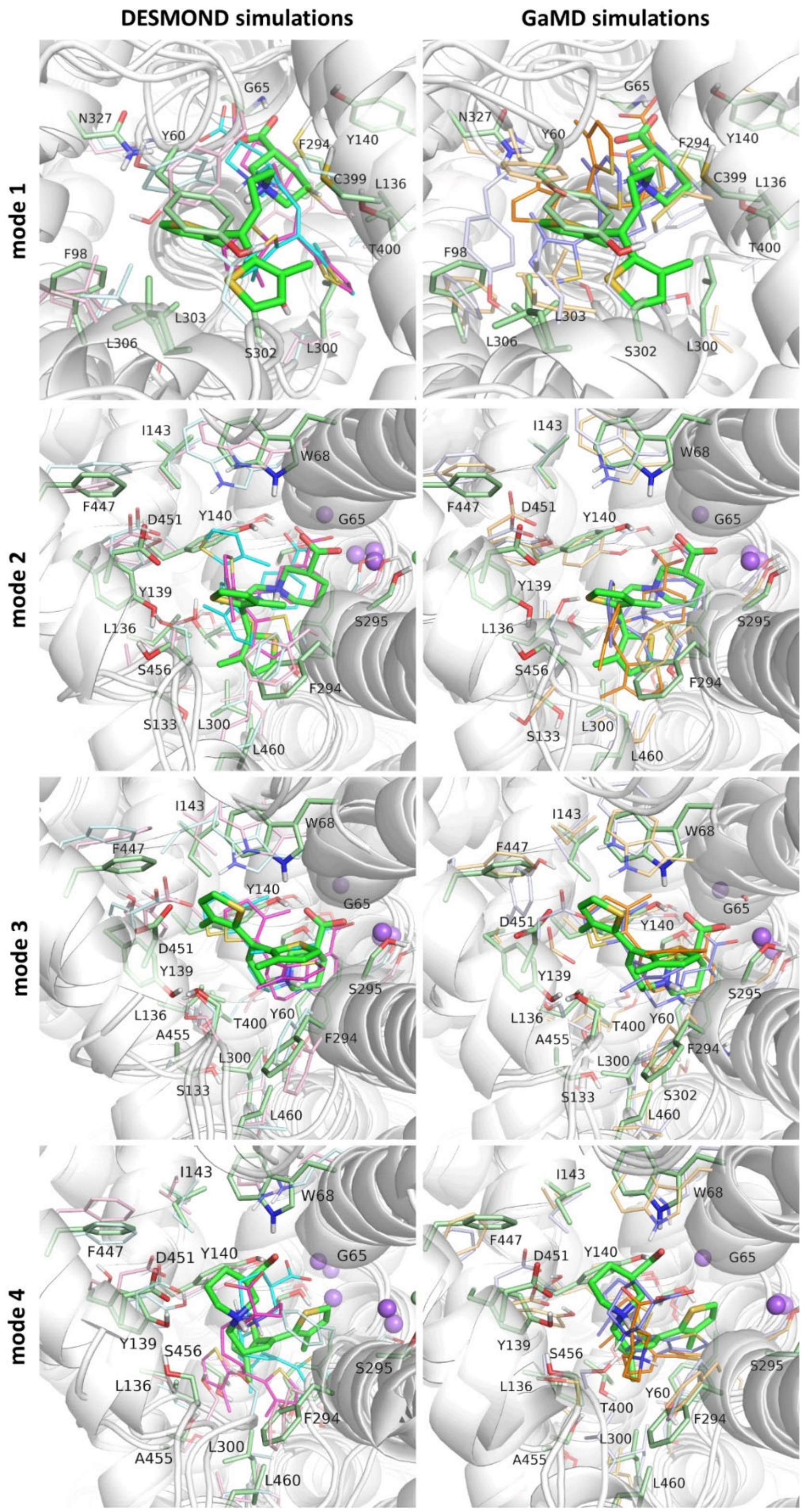

Figure S1. Investigated binding modes of tiagabine within hGAT-1, before (green) and after MD simulations (light blue – DESMOND MD 1; pink – DESMOND MD 2; light purple – GaMD 1; orange – GaMD 2).

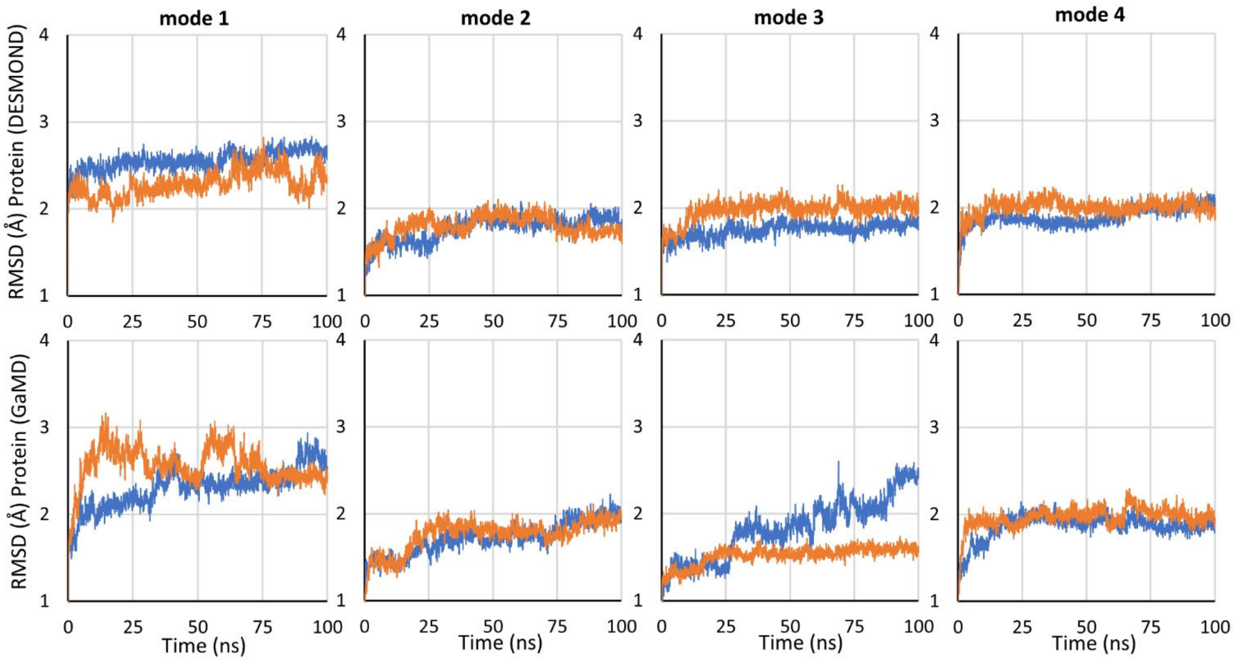

Figure S2. RMSD changes for hGAT-1 residues within 7 Å of tiagabine in different binding modes during molecular dynamics simulations carried out with DESMOND (classical MD) and NAMD (GaMD) programs.
